# First-Principles Study on the CO_2_ Reduction Reaction (CO_2_RR) Performance of h-BN-Based Single-Atom Catalysts Modified with Transition Metals

**DOI:** 10.3390/nano15080628

**Published:** 2025-04-20

**Authors:** Xiansheng Yu, Can Zhao, Qiaoyue Chen, Lai Wei, Xucai Zhao, Lili Zhang, Liqian Wu, Yineng Huang

**Affiliations:** 1 Xinjiang Laboratory of Phase Transitions and Microstructures in Condensed Matters, College of Physical Science and Technology, Yili Normal University, Yining 835000, China; yuxs997@sina.com (X.Y.); zhaocanqq@sina.com (C.Z.); chenqy0701@sina.com (Q.C.); lweiphy@sina.com (L.W.); zxc85619876@sina.com (X.Z.); 2 Yili Engineering Research Center of Green Silicon-Based Materials, Yining 835000, China; 3 Micro-Electronics Research Institute, Hangzhou Dianzi University, Hangzhou 310018, China; wulq@hdu.edu.cn; 4 National Laboratory of Solid State Microstructures, College of Physics, Nanjing University, Nanjing 210093, China

**Keywords:** first principles, h-BN, SACs, HER, CO_2_RR

## Abstract

The reasonable design of low-cost, high-activity single-atom catalysts (SACs) is crucial for achieving highly efficient electrochemical CO_2_RR. In this study, we systematically explore, using density functional theory (DFT), the performance of transition metal (TM = Mn, Fe, Co, Ni, Cu, Zn)-doped defect-type hexagonal boron nitride (h-BN) SACs TM@B_−1_N (B vacancy) and TM@BN_−1_ (N vacancy) in both CO_2_RR and the hydrogen evolution reaction (HER). Integrated crystal orbital Hamiltonian population (ICOHP) analysis reveals that these catalysts weaken the sp orbital hybridization of CO_2_, which promotes the formation of radical-state intermediates and significantly reduces the energy barrier for the hydrogenation reaction. Therefore, these theoretical calculations indicate that the Mn, Fe, Co@B_−1_N, and Co@BN_−1_ systems demonstrate excellent CO_2_ chemical adsorption properties. In the CO_2_RR pathway, Mn@B_−1_N exhibits the lowest limiting potential (*U_L_* = −0.524 V), and its higher d-band center (−0.334 eV), which aligns optimally with the adsorbate orbitals, highlights its excellent catalytic activity. Notably, Co@BN_−1_ exhibits the highest activity in HER, while *U_L_* is −0.217 V. Furthermore, comparative analysis reveals that Mn@B_−1_N shows 16.4 times higher selectivity for CO_2_RR than for HER. This study provides a theoretical framework for designing bifunctional SACs with selective reaction pathways. Mn@B_−1_N shows considerable potential for selective CO_2_ conversion, while Co@BN_−1_ demonstrates promising prospects for efficient hydrogen production.

## 1. Introduction

The continued rise in global energy demand and the accelerated consumption of fossil fuels [1,2] have become central challenges for the sustainable development of human society in the 21st century [3,4]. Although renewable energy technologies have advanced rapidly, their inherent intermittency severely limits large-scale application [5]. In this context, electrochemical CO_2_RR has gained considerable attention for its potential to convert greenhouse gases into high-energy-density hydrocarbons (such as CH_4_, C_2_H_4_, etc.), offering both environmental benefits and energy valorization [6,7,8]. The efficiency of this technology is largely dependent on catalyst design—highly active and selective electrocatalysts can significantly lower reaction overpotentials and precisely control product distribution [9,10,11].

Traditional noble metal catalysts, such as gold (Au) [12], palladium (Pd) [13], and platinum (Pt) [14], demonstrate exceptional catalytic activity in CO_2_RR. However, their widespread industrial application is severely hindered by high costs, limited availability, and inadequate thermal stability [15]. Since the introduction of SACs in 2011 [16], their atomically dispersed nature has garnered significant attention. Compared to conventional catalysts, SACs achieve nearly 100% atomic utilization [17] by anchoring transition metal (TM) atoms onto a support surface [18], dramatically improving economic efficiency. Theoretical studies suggest that electron transfer between the unoccupied d orbitals of TM atoms in SACs and the antibonding orbitals of CO_2_ effectively weakens the C=O bond, reducing its bond energy from 806 kJ·mol^−1^ to less than 750 kJ·mol^−1^ [19]. Additionally, the highly exposed active sites enhance intermediate adsorption and lower the reaction activation energy barrier, significantly boosting catalytic efficiency [20].

Two-dimensional materials, with their high specific surface area, ultra-fast carrier mobility, and abundant active sites at the interfaces [21], have emerged as ideal support platforms for TM single-atom catalysts in CO_2_RR. Among these, two-dimensional h-BN [22] forms a graphene-like honeycomb structure through sp^2^ hybridization. The strong covalent B-N bonds impart exceptional stability to the material, making it highly resistant to extreme conditions such as high temperatures and harsh acidic or basic environments [23]. By engineering B/N vacancies, strong coordination anchoring sites can be created on the surface of h-BN, allowing for high-density loading of TM single atoms. This facilitates an efficient catalytic interface for C=O bond activation and multi-electron transfer processes [24].

In recent years, significant advancements have been made in the study of h-BN-based SACs. For instance, Okello et al. [25] successfully developed functionalized h-BN heterostructures by incorporating transition metal nanoparticles and single-atom impurities into h-BN. Through a combination of scanning transmission electron microscopy (STEM) and density functional theory (DFT) analysis, they discovered that Ta, Co, Ni, and Ir single atoms preferentially adsorb at the N-top site of h-BN. The strong agreement between experimental and theoretical results offers valuable insights for catalyst design. Chen et al. [26] loaded Pd nanoparticles (NPs) onto the surface of h-BN and found that the strong interaction between h-BN and Pd could tune the d-band center of Pd, optimizing the adsorption energy of reaction intermediates and significantly enhancing electrocatalytic performance. Li et al. [27], through DFT screening, discovered that Fe-doped h-BN (Fe@BN) exhibits the lowest barrier (0.52 eV) and the highest exothermic energy (4.37 eV) for CO oxidation, demonstrating exceptional catalytic potential. Additionally, Wang et al. [28] confirmed that Cu@O_1_N_2_-BN catalysts show a very low rate-limiting step energy barrier of 0.46 eV in the direct methane-to-methanol conversion reaction. Zhao et al. [29] found that Mo@BN monolayers exhibit an overpotential of only 0.19 V for nitrogen reduction reaction (NRR), highlighting their outstanding catalytic activity. Konopatsky A et al. [30] reported the Ag-doped layered ferrihydrite (Fh)/h-BN heterostructures synthesized via the solvent thermal synthesis method exhibited a 2.5-fold enhancement in CO_2_ conversion efficiency compared to the initial value, with olefin selectivity increasing from 0.31% to 0.88%.

Although h-BN-based SACs have demonstrated exceptional performance in areas such as HER [31], oxygen evolution reaction (OER) [32], and nitrogen reduction reaction (NRR) [33,34], and CO_2_RR, this study will conduct an in-depth theoretical investigation into the mechanism of CO_2_RR on h-BN-based SACs. Experimental studies have demonstrated that Fe-doped h-BN catalysts exhibit promising CO_2_ conversion efficiency; however, their stability remains suboptimal, necessitating further exploration of the influence of h-BN defect types (e.g., B vacancies versus N vacancies) on catalytic pathways and the extension of research to other transition metal/h-BN systems [35]. To address these gaps, we systematically explore the CO_2_RR performance of Fe and other transition metals within the same period (Mn, Co, Ni, Cu, and Zn) embedded in defective h-BN (TM@B_−1_N and TM@BN_−1_) through DFT calculations. The focus is on analyzing structural stability, electronic properties, and the kinetics of C=O bond activation, with the goal of providing a theoretical foundation for designing defected h-BN-based SACs and optimizing their application in CO_2_RR and other related reactions.

## 2. Materials and Methods

In this study, first-principles calculations based on DFT are performed using the Vienna Ab Initio Simulation Package (VASP 6.3.0) software [36]. The Perdew–Burke–Ernzerhof (PBE) exchange-correlation functional [37] within the generalized gradient approximation (GGA) framework is applied, with a plane-wave cutoff energy set to 500 eV. The Brillouin zone integration is carried out using the Monkhorst–Pack method [38], generating a 3 × 3 × 1 k-point mesh. The energy convergence criterion is set to 1 × 10^−5^ eV/atom, and the Hellmann–Feynman force convergence criterion during structural relaxation is set to less than 0.05 eV/Å for each atom. To accurately capture interlayer van der Waals interactions, the DFT-D3 method [39] is used for van der Waals corrections. In this study, charge transfer between the catalyst and the adsorbates is analyzed based on quantum theory of atoms in molecules (QTAIM) [40] charge calculations, using the Bader charge analysis method [41]. The atomic electronic configurations for the elements involved in this study are as follows: B: 2s^2^2p^1^, N: 2s^2^2p^3^, Mn: 3d^5^4s^2^, Fe: 3d^6^4s^2^, Co: 3d^7^4s^2^, Ni: 3d^8^4s^2^, Cu: 3d^10^4s^1^, Zn: 3d^10^4s^2^.

In this study, the pristine hexagonal boron nitride (h-BN) crystal structure serves as the foundational model. It belongs to the P6_3_/mmc space group (No. 194) with lattice parameters of *a* = *b* = 2.510 Å, *c* = 6.690 Å, *α* = *β* = 90°, and *γ* = 120° [42].

## 3. Results

### 3.1. Subsection

**Figure 1:**
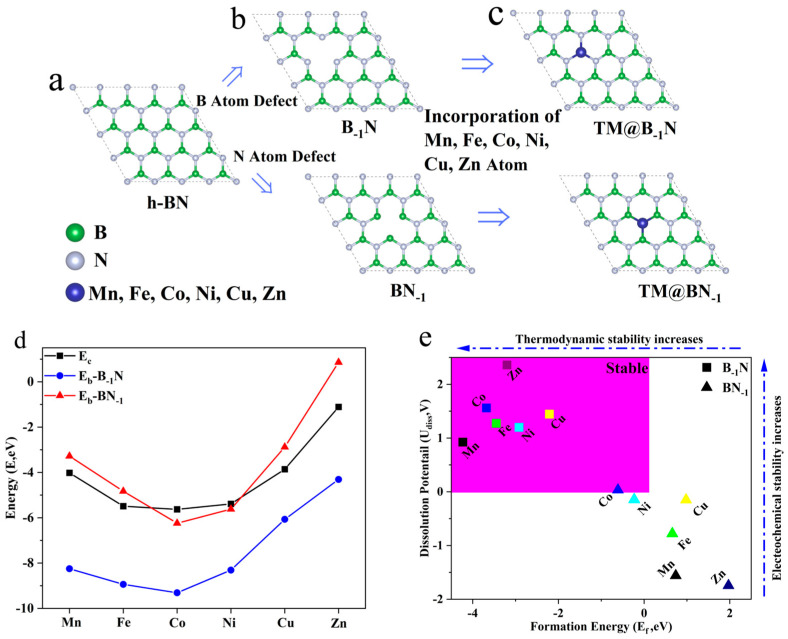
(**a**) Structure of h-BN; (**b**) structure of B_−1_N and BN_−1_; (**c**) structure of TM@B_−1_N and TM@BN_−1_; (**d**) binding energy and cohesive energy in TM@B_−1_N and TM@BN_−1_; (**e**) formation energy and dissolution potential of metal atoms in TM@B_−1_N and TM@BN_−1_.

To construct the monolayer h-BN model, the structure is exfoliated along the (001) plane, and a 20 Å vacuum layer is introduced in the vertical direction to eliminate periodic interactions. After geometric optimization (Figure 1a), the equilibrium lattice parameters of the h-BN monolayer converge to *a* = *b* = 2.464 Å and *c* = 19.447 Å. To further reduce edge effects, a 3 × 3 × 1 supercell is constructed to establish a periodic system (Figure 1b). A single B or N atom is selectively removed from the center of the supercell, resulting in B single-vacancy (B_−1_N) and N single-vacancy (BN_−1_) defect structures, respectively [43].

To construct the single-atom catalyst model (Figure 1c), transition metal atoms TM are embedded into the defect sites with sub-angstrom precision (Δ*z* = 0.1 Å). In the B-1N system, the metal atom coordinates with three neighboring N atoms, forming an M-N_3_ active center (denoted as TM@B_−1_N). Conversely, in the BN_−1_ system, the metal atom bonds with three B atoms, creating an TM-B_3_ active site (denoted as TM@BN_−1_). This asymmetric embedding effectively breaks the intrinsic symmetry of the support structure, establishing an ideal model system for exploring metal–support synergistic effects in single-atom catalysts.

To assess the single-atom dispersion stability of transition metal atoms at defect sites in h-BN, this study evaluates the catalytic systems’ stability using two key criteria: binding energy (*E_b_*) and cohesive energy (*E_c_*). The binding energy (*E_b_*) quantifies the strength of interaction between the metal atom and the support, calculated as [44]:

(1) $$E_{b}=E_{total}-E_{def}-E_{M}$$

Meanwhile, the cohesive energy (*E_c_*) represents the metal atom’s tendency to aggregate, defined as [45]:

(2) $$E_{c}=E_{bulk}-E_{M}$$

where *E_total_*, *E_def_*, *E_M_*, and *E_bulk_* denote the total energy of the loaded system, the energy of the defective support, the energy of an isolated metal atom, and the per atom energy in the bulk metal phase, respectively. More negative values of *E_b_* and *E_c_* indicate stronger binding interactions.

As illustrated in Figure 1d, the condition *E_b_* < *E_c_* < 0 signifies that metal–support interactions outweigh metal–metal aggregation tendencies, ensuring the stability of single-atom dispersion. The results reveal that in the B-vacancy system (TM@B_−1_N), all TMs satisfy *E_b_* < *E_c_*, demonstrating the robust anchoring capability of B vacancies for transition metals. Conversely, in the N-vacancy system (TM@BN_−1_), only Co and Ni meet the *E_b_* < *E_c_* criterion, while Mn, Fe, Cu, and Zn exhibit *E_b_* > *E_c_*, indicating a higher propensity to aggregate into nanoclusters at N-vacancy sites.

To comprehensively assess the stability of the catalytic system, this study further evaluates the feasibility of candidate systems based on two additional criteria: thermodynamic formation energy (*E_f_*) and electrochemical dissolution potential (*U_diss_*). These criteria are defined as follows [46]:

(3) $$E_{f}=E_{total}-E_{def}-E_{bulk}$$

(4) $$U_{diss}=U_{diss}^{0}(metal,bulk)-\frac{Ef}{ne}$$

where $U_{diss}^{0}$ (*metal*, *bulk*) represents the standard dissolution potential of the bulk metal, and n_e_ denotes the number of electrons involved in the dissolution process. As illustrated in Figure 1e, when *E_f_* < 0, the metal–support composite system exhibits lower energy than its separated components, indicating potential synthetic feasibility in experiments [47]. Moreover, a positive dissolution potential (*U_diss_* > 0) suggests that the catalyst can resist metal dissolution under reductive conditions [48]. The computational results reveal that all TM@B_−1_N systems satisfy *E_f_* < 0 and *U_diss_* > 0, confirming both synthetic feasibility and electrochemical stability. The Co@BN_−1_ system meets the two stability criterion which are *E_f_* < 0 and *U_diss_* > 0. However, the Ni@BN_−1_ system fails to satisfy both structural stability conditions simultaneously, particularly due to *U_diss_* < 0, which suggests a propensity for structural reconstruction during the catalytic process. Consequently, this system will not be taken into account in subsequent studies. Therefore, all TM@B_−1_N and Co@BN_−1_ systems demonstrate both thermodynamic and electrochemical stability, making them promising candidates for catalytic applications.

According to Table S2, in TM@B_−1_N systems, a shorter TM-N bond length correlates with a lower *E_b_*, indicating greater stability. For Mn@B_−1_N and Cu@B_−1_N, although the difference in TM-N bond lengths is merely 0.00113 Å, the *E_b_* differs by as much as 2.18 eV. This suggests that nitrogen atoms exhibit stronger bonding interactions with TM atoms that possess incompletely filled penultimate electron shells. Moreover, in both Co@B_−1_N and Co@BN_−1_ configurations, comparisons of TM-B/N bond lengths and *E_b_* consistently demonstrate a stronger affinity between TM atoms and nitrogen atoms within the substrate.

**Figure 2:**
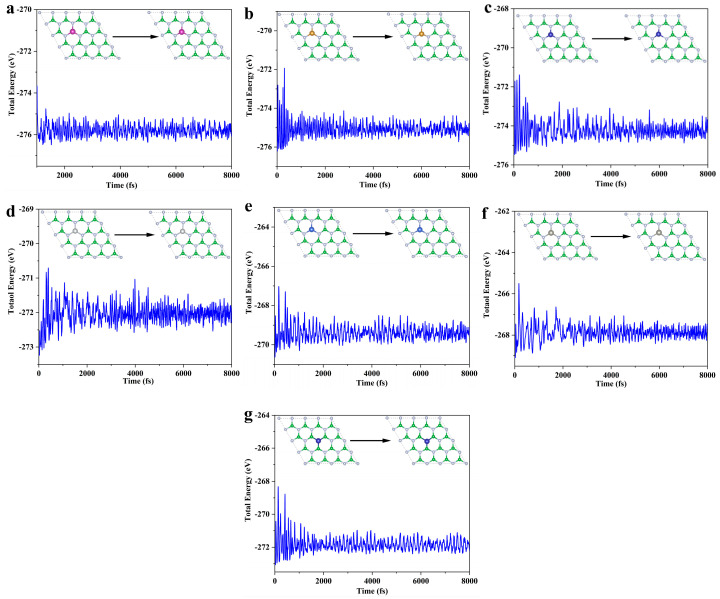
SACs AIMD: (**a**) Mn@B_−1_N; (**b**) Fe@B_−1_N; (**c**) Co@B_−1_N; (**d**) Ni@B_−1_N; (**e**) Cu@B_−1_N; (**f**) Zn@B_−1_N; (**g**) Co@BN_−1_.

Building on the thermodynamic and electrochemical stability assessments, this study selects the TM@B_−1_N and Co@BN_−1_ systems for ab initio molecular dynamics (AIMD) [49] simulations to further evaluate their dynamic stability. The simulations are conducted at 300 K (room temperature) for a total duration of 8 ps (8000 steps, with a time step of 1 fs). As illustrated in Figure 2, the dynamic behavior analysis of all systems indicates minimal fluctuations in TM-N/B bond lengths, with no evidence of metal–support bond dissociation. Furthermore, the total energy fluctuations gradually stabilize, satisfying the criteria for dynamic stability [50]. These results confirm that the selected SACs possess both structural robustness and dynamic stability under ambient conditions, making them promising candidates for practical catalytic applications.

### 3.2. CO_2_ Adsorption and Activation

In this study, the CO_2_ adsorption and activation mechanisms are systematically explored through a combination of density of states (DOS) analysis and adsorption calculations. As shown in Figure S1, transition metal doping induces significant modulation of the electronic structure. Specifically, the reduced bandgap in the TM@B_−1_N and Co@BN_−1_ systems enhances their conductivity [51], thereby promoting charge transfer during the catalytic process.

The selectivity of CO_2_ electrochemical reduction pathways (producing CH_4_, CH_3_OH, or CO) is strongly influenced by its adsorption configuration and activation degree [52]. To quantify the interaction strength between the catalyst and reactant, the adsorption energy (*E_ads_*) is calculated as follows:

(5) $$E_{ads}=E_{total-CO_{2}}-E_{total}-E_{CO_{2}}$$

where *E_total-CO_*_2_, *E_total_*, and *E_CO_*_2_ represent the total energy of the adsorption system, the intrinsic energy of the catalyst, and the energy of a free CO_2_ molecule, respectively [53].

**Figure 3:**
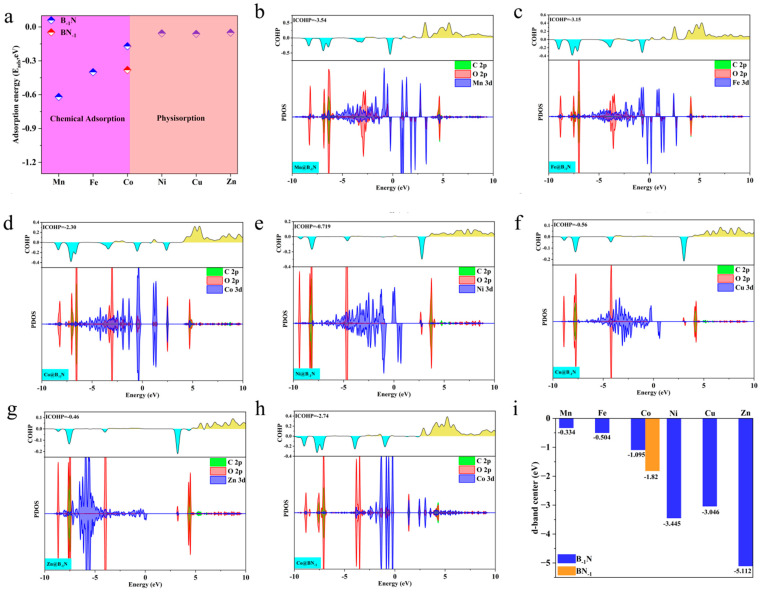
(**a**) Adsorption energies of CO_2_ on TM@B_−1_N and Co@BN_−1_ surfaces; (**b**–**h**) PDOS and the COHPs of CO_2_ on TM@B_−1_N and Co@BN_−1_ surfaces; the bonding and antibonding states in COHP are depicted by cyan and yellow, respectively. (**i**) The d-band center of TM@B_−1_N and Co@BN_−1_.

As shown in Figure 3a, Mn@B_−1_N, Fe@B_−1_N, Co@B_−1_N, and Co@BN_−1_ exhibit significantly exothermic adsorption, indicating strong chemisorption of CO_2_. In contrast, Ni@B_−1_N, Cu@B_−1_N, and Zn@B_−1_N show adsorption energies close to zero, suggesting weak physisorption. The electronic interaction mechanisms were systematically investigated through electron localization function (ELF) analysis (Figure S2). For both Mn/Fe/Co@B_−1_N and Co@BN_−1_ configurations, the ELF values spanning the full spectrum from 0 to 1 between CO_2_ molecules and catalytic active sites reveal significant charge redistribution patterns. This complete coverage of ELF values across the theoretical range (η = 0.4) provides conclusive evidence for the establishment of robust chemical bonding interactions, confirming the coexistence of covalent character and strong ionic bonding components in these catalyst–CO_2_ complexes. For the Ni/Cu/Zn@B_−1_N system, ELF analysis that the computed values persistently cluster near the bound (η ≈ 0), there is no exchange between the components. Electrons are localized on the components of the system. This corresponds to the interaction of closed shells. In the case of (d, e, f), there is an interaction of closed shells, i.e., van der Waals bonds, providing conclusive evidence of van der Waals-dominated interactions characterized by weak electronic coupling. These distinct adsorption modes directly impact catalytic performance. Only chemisorbed CO_2_ undergoes molecular bending and C=O bond elongation, facilitating effective charge transfer and molecular activation—critical prerequisites for subsequent reduction reactions.

To further clarify the electronic nature of CO_2_ activation in chemisorption and physisorption, this study employs projected density of states (PDOS) and crystal orbital Hamilton population (COHP) analyses to investigate the interaction mechanisms between metal atoms and the adsorbed CO_2_ molecule. As illustrated in Figure 3b–d,h, chemisorbed systems (Mn/Fe/Co@B_−1_N and Co@BN_−1_) exhibit pronounced hybridization peaks near the Fermi level, arising from the interaction between metal d orbitals and the antibonding π* orbitals of CO_2_. This strong orbital coupling induces molecular bending and C=O bond elongation while facilitating a dual-channel charge transfer mechanism: the unoccupied d orbitals of the metal capture electrons from the bonding orbitals of CO_2_, while the occupied d orbitals donate electrons into the CO_2_ antibonding orbitals. Consequently, the CO_2_ molecule acquires radical-like intermediate characteristics, enhancing its activation potential.

ICOHP analysis further substantiates these findings. As shown in Figure 3b–d,h, the chemisorbed systems exhibit strongly negative ICOHP values (Mn@B_−1_N: −3.54 eV, Fe@B_−1_N: −3.15 eV, Co@B_−1_N: −2.30 eV, Co@BN_−1_: −2.74 eV), signifying deep orbital coupling between the metal d orbitals and the CO_2_ π* orbitals [54]. This robust interaction induces orbital hybridization near the Fermi level with the s and p orbitals of C and O, weakening the sp hybridization strength of CO_2_ and lowering the C=O dissociation energy barrier. Conversely, Figure 3e–g illustrate that in the physisorbed systems (Ni/Cu/Zn@B_−1_N), the overlap between metal d orbitals and CO_2_ π* orbitals is negligible (< 0.05), and CO_2_ retains its linear configuration due to van der Waals interactions alone. The weak ICOHP values (Ni@B_−1_N: −0.72 eV, Cu@B_−1_N: −0.56 eV, Zn@B_−1_N: −0.46 eV) further confirm the feeble nature of this interaction, aligning with the minimal charge transfer (ΔQ < 0.02 e^−^, Table S2). These findings highlight that the strength of d-π* orbital coupling, by regulating electronic state occupancy, plays a decisive role in determining the degree of CO_2_ activation and ultimately dictates the selectivity of subsequent reduction reaction pathways.

The d-band center, a key descriptor of the electronic structure of transition metal surfaces, is defined as the weighted average energy of the metal d-orbital density of states. It is calculated using the following equation [55]:

(6) $$\varepsilon =\int_{-\infty }^{+\infty }n_{d}(\varepsilon )\varepsilon d_{\varepsilon }/\int_{-\infty }^{+\infty }n_{d}(\varepsilon )d_{\varepsilon }$$

A higher d-band center value indicates that the d-orbital energy levels shift closer to the Fermi level, thereby enhancing orbital hybridization with adsorbed species [56]. As shown in Figure 3i, the d-band center values of the TM@B_−1_N and Co@BN_−1_ systems follow the order: Mn@B_−1_N (−1.8 eV) > Fe@B_−1_N (−2.1 eV) > Co@BN_−1_ (−2.3 eV) > Co@B_−1_N (−2.5 eV) > Ni/Cu/Zn@B_−1_N (−3.2 to −3.6 eV). This trend strongly correlates with the observed decrease in *E_ads_* and ICOHP. In systems with lower d-band centers (Ni/Cu/Zn@B_−1_N), the d-orbital energy levels are significantly lower than the CO_2_ π* orbitals, preventing effective disruption of CO_2_ sp-hybridized bonds and resulting in weak physisorption via van der Waals interactions. In contrast, catalysts with higher d-band centers (Mn/Fe/Co-based systems) promote CO_2_ activation through strong d–π* antibonding orbital coupling. The predicted catalytic activity follows the order: Mn@B_−1_N > Fe@B_−1_N > Co@BN_−1_ > Co@B_−1_N, providing an electronic structure-based framework for selecting optimal reaction pathways [57].

### 3.3. CO_2_ Reduction Reaction (CO_2_RR) Performance

This study utilizes the computational hydrogen electrode (CHE) model proposed by Nørskov et al. [58] to assess the thermodynamic feasibility of CO_2_ electrochemical reduction pathways by computing the Gibbs free energy change (Δ*G*). The calculation is expressed as [59]:

(7) $$G=E_{H}+E_{ZPE}-TS$$

where *E_H_* is the total energy of the system, *E_ZPE_* represents the zero-point energy, and *S* corresponds to the entropy change, determined under a fixed-atom model for the catalyst.

By analyzing the Δ*G* for the Mn@B_−1_N, Fe@B_−1_N, Co@B_−1_N, and Co@BN_−1_ catalytic systems (Figure S3), the potential determining step (*PDS*) for each reaction pathway is identified. According to the Brønsted–Evans–Polanyi [60] relationship, reaction steps with lower Δ*G* values correspond to lower activation energy barriers, thereby controlling the reaction progression. Key pathway analysis reveals the following: CO hydrogenation (*CO + H⁺ + e⁻ → *CHO/*COH): all catalysts preferentially form the *CHO intermediate. CHO hydrogenation (*CHO + H⁺ + e⁻ → *CHOH/*CH_2_O): Mn@B_−1_N and Co@BN_−1_ favor *CHOH formation (Δ*G* = 0.42 eV). In the Fe@B_−1_N pathway, OCHH forms first (Δ*G* = 0.77 eV); however, its subsequent *OH desorption barrier (1.2 eV) is significantly lower than that of the *CHOH pathway (1.8 eV), making OCHH the preferred route. Co@B_−1_N follows a distinct mechanism: the *COH → *CHOH step presents a prohibitively high Δ*G* of 3.73 eV, whereas *OCHH formation is more energetically favorable with a maximum Δ*G* of 2.15 eV, confirming *OCHH as the preferred intermediate.

These results indicate that Mn-/Fe-based catalysts with higher d band centers stabilize the *CHO intermediate, thereby lowering the reduction energy barrier. In contrast, Co-based systems exhibit pathway selectivity differences due to geometric steric effects, providing theoretical guidance for tuning multi-carbon product formation.

**Figure 4:**
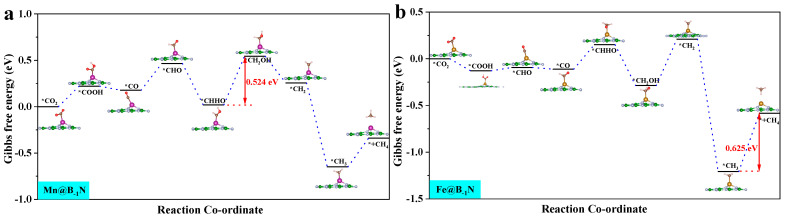

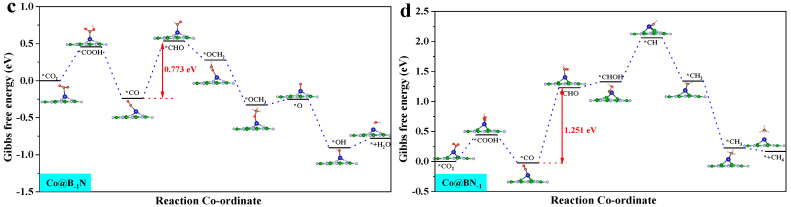
Gibbs free energy step diagram of single-atom catalyst CO_2_RR: (**a**) Mn@B_−1_N; (**b**) Fe@B_−1_N; (**c**) Co@B_−1_N; (**d**) Co@BN_−1_. The “*” in the figure indicates the catalyst.

*U_L_* represents the minimum overpotential required to drive the *PDS* in a catalytic reaction. It is calculated as [61]:

(8) $$UL=-\frac{\Delta G_{PDS}}{e}$$

where Δ*G_PDS_* is the Gibbs free energy change associated with the *PDS*, and e is the elementary charge. A more negative *U_L_* signifies a higher thermodynamic energy barrier, indicating reduced catalytic activity [62]. Figure 4 presents the optimal CO_2_ reduction pathways for Mn@B_−1_N, Fe@B_−1_N, Co@B_−1_N, and Co@BN_−1_ catalysts, with their *U_L_* values ranked as follows: Mn@B_−1_N (−0.524 V) > Fe@B_−1_N (−0.612 V) > Co@B_−1_N (−0.683 V) > Co@BN_−1_ (−0.718 V). For the Mn@B_−1_N system, the *PDS* is *CHHO + (H⁺ + e⁻) + H_2_O → *CH_2_OH + H_2_O, which corresponds to the lowest *U_L_* value, indicating the highest catalytic activity. A comprehensive comparative analysis (Table 1) reveals a monotonic decrease in *U_L_* values that exhibits strong correlation with the sequential reduction in both ICOHP parameters and d band center positions, demonstrating that the energy alignment of metal d-orbitals orchestrates catalytic performance through a dual-parameter modulation mechanism: simultaneously regulating activation kinetics through ICOHP and d band center [63].

### 3.4. Competition Between CO_2_RR and HER

**Figure 5:**
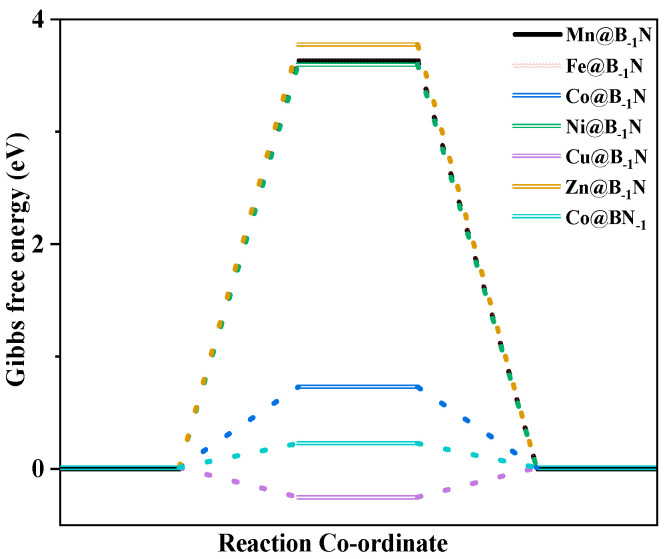
Diagram of the HER Gibbs free energy step of SACs.

To elucidate the competitive selectivity between CO_2_RR and the HER on TM@B_−1_N and Co@BN_−1_ catalysts, this study systematically evaluates *U_L_* and the Gibbs free energy change (Δ*G*) in key intermediates to uncover the underlying mechanism [64,65]. HER performance analysis (Figure 5) reveals that Co@BN_−1_ demonstrates the highest hydrogen evolution activity (*U_L_* = −0.217 V), significantly surpassing other systems (Mn@B_−1_N: −3.631 V, Fe@B_−1_N: −3.635 V, Co@B_−1_N: −0.721 V, Cu@B_−1_N: −0.263 V, Zn@B_−1_N: −3.775 V). By comparing with Table S4, it is evident that the charge transfer between Co@BN-1 and the adsorbed H is more efficient. This superior HER activity in Co@BN_−1_ can be attributed to the strong hybridization between Co 3d orbitals and adjacent B 2p orbitals near the Fermi level, which markedly enhances proton (H^+^ + e^−^) adsorption and promotes the Volmer–Heyrovsky reaction pathway.

By analyzing Table 2 in conjunction with the Brønsted–Evans–Polanyi relationship, it is evident that the Mn/Fe/Co@B_−1_N systems favor CO_2_ reduction, as the Gibbs free energy change for CO_2_ activation (G*COOH) is significantly lower than that for HER (ΔG*H). In contrast, Co@BN_−1_ exhibits dominant HER activity due to its higher ΔG*COOH (0.28 eV) compared to ΔG*H (−0.15 eV), making hydrogen evolution energetically more favorable. This competitive mechanism underscores the critical role of the synergy between transition metal d orbitals and the coordination environment of the support in dictating CO_2_RR/HER pathway selectivity. By fine-tuning the relative stability of *COOH and *H intermediates, this synergy provides a precise means of steering reaction outcomes. These insights offer a dual-regulation strategy—integrating electronic structure engineering and thermodynamic modulation—for the rational design of highly selective electrocatalysts.

## 4. Conclusions

This study systematically elucidates the competitive regulation mechanisms of CO_2_RR and HER in transition metal-embedded defective h-BN systems (TM@B_−1_N and TM@BN_−1_). Key findings include: (1) optimal CO_2_RR activity: the Mn@B_−1_N system exhibits exceptional CO_2_RR performance, with its low limiting potential (*U_L_* = −0.524 V), high d-band center (−0.334 eV), and strong antibonding orbital coupling with CO_2_ (ICOHP = −3.54 eV) synergistically reducing the reaction energy barrier; (2) HER selectivity control: the Co@BN_−1_ system demonstrates HER activity (*U_L_* = −0.217 V) comparable to commercial Pt-based catalysts, driven by strong hybridization between Co-3d and B-2p orbitals near the Fermi level, which facilitates proton adsorption and Heyrovsky pathway kinetics; (3) reaction pathway competition: Mn/Fe/Co@B_−1_N systems activate CO_2_ via d-π* orbital coupling to disrupt sp hybridization, forming radical intermediates, whereas Ni/Cu/Zn@B_−1_N systems only adsorb linear CO_2_ through van der Waals interactions, resulting in ineffective activation; (4) stability and design strategy: all B-vacancy systems satisfy thermodynamic stability criteria (*E_b_* < *E_c_*, *U_diss_* > 0), while only Co@BN_−1_ remains stable among N-vacancy configurations. Based on these insights, we propose an application-targeted design principle: Mn@B_−1_N with high d-band center and strong orbital coupling is optimal for CO_2_ conversion; Co@BN_−1_ is preferred for hydrogen production; and the balance between CO_2_RR/HER pathways can be engineered by tailoring defect types (B/N vacancies) and metal d-orbital energy levels.

The DFT calculations in this study reveal that Mn@B_−1_N is the most suitable catalyst for CO_2_ reduction in h-BN-based single-atom catalysts, with a limiting potential of −0.524 V, while Co@BN_−1_ promotes hydrogen evolution with a limiting potential of −0.217 V, providing structural selection criteria for experimental synthesis. By controlling the type of metal (such as Mn or Co) and the type of coordinating defects (B or N vacancies), we can directionally optimize the hybridization strength between the d orbitals and the substrate, balancing the competition between CO_2_ activation and hydrogen adsorption, thereby guiding the preparation of highly selective bifunctional catalysts.

## Figures and Tables

**Table 1:** Comparative table of catalytic activity results.

	CO_2_ Adsorption	*U_L_* of CO_2_RR (V)	*U_L_* of HER (V)	d Band Center (eV)	ICOHP (eV)	Stability	CO_2_RR vs. HER
Mn@B_−1_N	chemisorption	−0.524	−3.631	−0.334	−3.54	stable	CO_2_RR (16.4 times)
Fe@B_−1_N	chemisorption	−0.625	−3.635	−0.504	−3.15	stable	CO_2_RR
Co@B_−1_N	chemisorption	−0.773	−0.721	−1.095	−2.30	stable	CO_2_RR
Ni@B_−1_N	physisorption	/	−3.599	−3.445	−0.72	stable	No CO_2_RR activity
Cu@B_−1_N	physisorption	/	−0.263	−3.046	−0.56	stable	No CO_2_RR activity
Zn@B_−1_N	physisorption	/	−3.775	−5.112	−0.46	stable	No CO_2_RR activity
Co@BN_−1_	chemisorption	−1.251	−0.217	−1.82	−2.74	stable	HER

**Table 2:** Gibbs free energy changes (Δ*G*) in initial protonation of CO_2_RR vs. HER on TM@B_−1_N and TM@BN_−1_ surfaces.

	Δ*G*(*H)/eV	Δ*G*(*COOH)/eV
Mn@B_−1_N	3.631	0.221
Fe@B_−1_N	3.635	−0.129
Co@B_−1_N	0.721	0.456
Ni@B_−1_N	3.599	/
Cu@B_−1_N	−0.263	/
Zn@B_−1_N	3.775	/
Co@BN_−1_	0.217	0.445

## Data Availability

The original contributions presented in this study are included in the article, and further inquiries can be directed to the corresponding authors.

## Supplementary Materials

The following supporting information can be downloaded at: https://www.mdpi.com/article/10.3390/nano15080628/s1, Table S1: *E_b_*, *E_C_*, *E_bulk_*, *U_diss_* of metals, *N_e_* during the dissolution and *E_f_*; Table S2: The bond length of TM-N/B in TM@B_−1_N and Co@B_−1_N. Figure S1: Density of States (DOS) of TM (Mn, Fe, Co, Ni, Cu, Zn)@B_−1_N and Co@BN_−1_. Figure S2: ELF values on the (–1, 1, –1) isosurface of the SCAs: (a) Mn@B_−1_N; (b) Fe@B_−1_N; (c) Co@B_−1_N; (d) Ni@B_−1_N; (e) Cu@B_−1_N; (f) Zn@B_−1_N; (g) Co@BN_−1_. Table S3: The number of charges transferred by a monatomic catalyst to CO_2_. Table S4: The number of charges transferred by SACs to H. Figure S3: Seven specific reaction pathways for the production of CH_4_ through the CO_2_RR (Carbon Dioxide Reduction Reaction), Gibbs free energy step diagram of single-atom catalyst CO_2_RR: (a) Mn@B_−1_N; (b) Fe@B_−1_N; (c) Co@B_−1_N; (d) Co@BN_−1_.
