# Supplementary material for: First-Principles Study on the CO2 Reduction Reaction (CO2RR) Performance of h-BN-Based Single-Atom Catalysts Modified with Transition Metals
Source: Nanomaterials (Basel). 2025 Apr 20;15(8):628. doi: 10.3390/nano15080628 (PMC12029760; doi:10.3390/nano15080628)
Supplement: Supplementary file 1 [file nanomaterials-15-00628-s001.zip › nanomaterials-3580435-supplementary.pdf]

First-Principles Study on the CO_2 Reduction Reaction (CO_2RR) Performance of h-BN-Based Single-Atom Catalysts Modified with Transition Metals

Table S1 Binding energy (E_b), Cohesive energy (E_C), Total energy of metal atoms in their bulk phase (E_{bulk}), dissolution potential (U_{diss}) of metals, number of transferred electrons (N_e) during the dissolution and Formation energy (E_f).

	E_C (eV)	N_e	U^0_{diss}(V)	E_{bulk} (eV)	E_b (eV)	E_f (eV)	U_{diss} (V)
**Mn@B-1N**	**-4.02**	**2**	**-1.19**	**-9.16**	**-8.25**	**-4.23**	**0.925**
**Fe@B-1N**	**-5.49**	**2**	**-0.45**	**-8.46**	**-8.94**	**-3.45**	**1.275**
**Co@B-1N**	**-5.63**	**2**	**-0.28**	**-7.11**	**-9.31**	**-3.68**	**1.56**
**Ni@B-1N**	**-5.39**	**2**	**-0.26**	**-5.78**	**-8.31**	**-2.92**	**1.2**
**Cu@B-1N**	**-3.86**	**2**	**0.34**	**-4.10**	**-6.07**	**-2.21**	**0.845**
**Zn@B-1N**	**-1.11**	**2**	**-0.76**	**-1.27**	**-4.31**	**-3.2**	**1.34**
**Mn@BN-1**	**-4.02**	**2**	**-1.19**	**-9.16**	**-3.28**	**0.74**	**-1.56**
**Fe@BN-1**	**-5.49**	**2**	**-0.45**	**-8.46**	**-4.83**	**-3.45**	**1.275**
**Co@BN-1**	**-5.63**	**2**	**-0.28**	**-7.11**	**-6.24**	**-0.61**	**0.025**
**Ni@BN-1**	**-5.39**	**2**	**-0.26**	**-5.78**	**-5.62**	**-0.23**	**-0.145**
**Cu@BN-1**	**-3.86**	**2**	**0.34**	**-4.10**	**-2.88**	**0.98**	**-0.15**
**Zn@BN-1**	**-1.11**	**2**	**-0.76**	**-1.27**	**0.86**	**1.97**	**-1.745**

Table S2 The bond length of TM-N/B in TM@B-1N and Co@B-1N

	TM-N/B (Å)
Mn@B-1N	1.83746
Fe@B-1N	1.83729
Co@B-1N	1.75797
Ni@B-1N	1.83737
Cu@B-1N	1.83859
Zn@B-1N	1.85163
Co@BN-1	1.85371

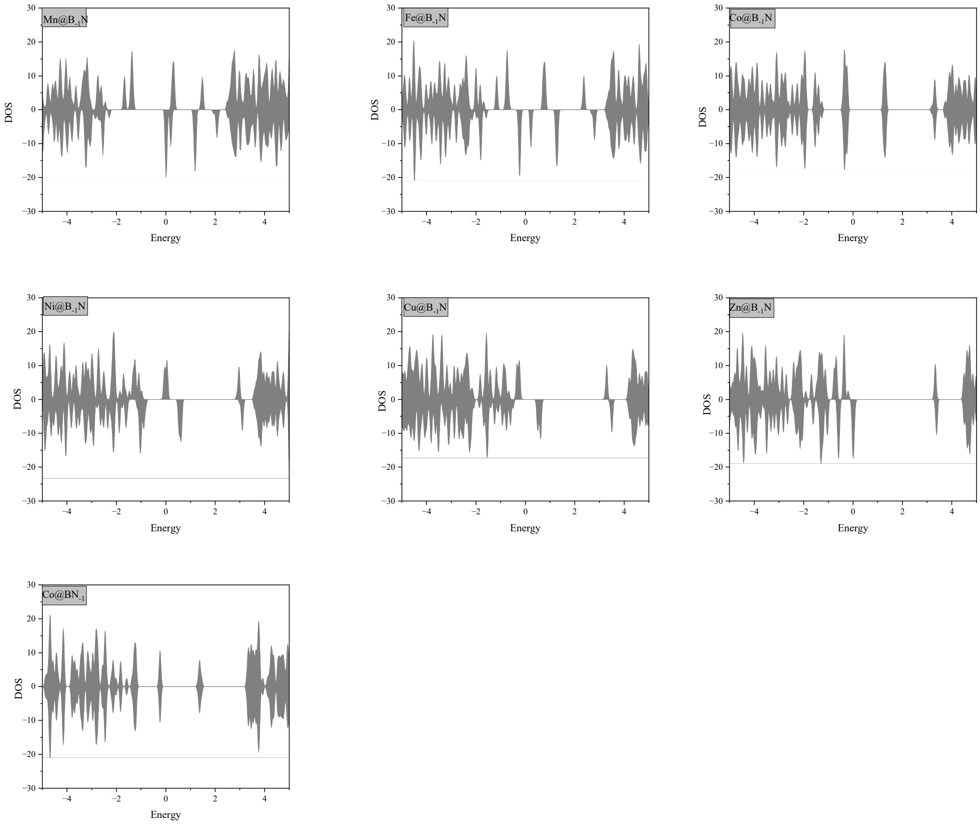

Figure S1 Density of States (DOS) of TM (Mn, Fe, Co, Ni, Cu, Zn)@$B_{-1}N$ and Co@BN_{-1}.

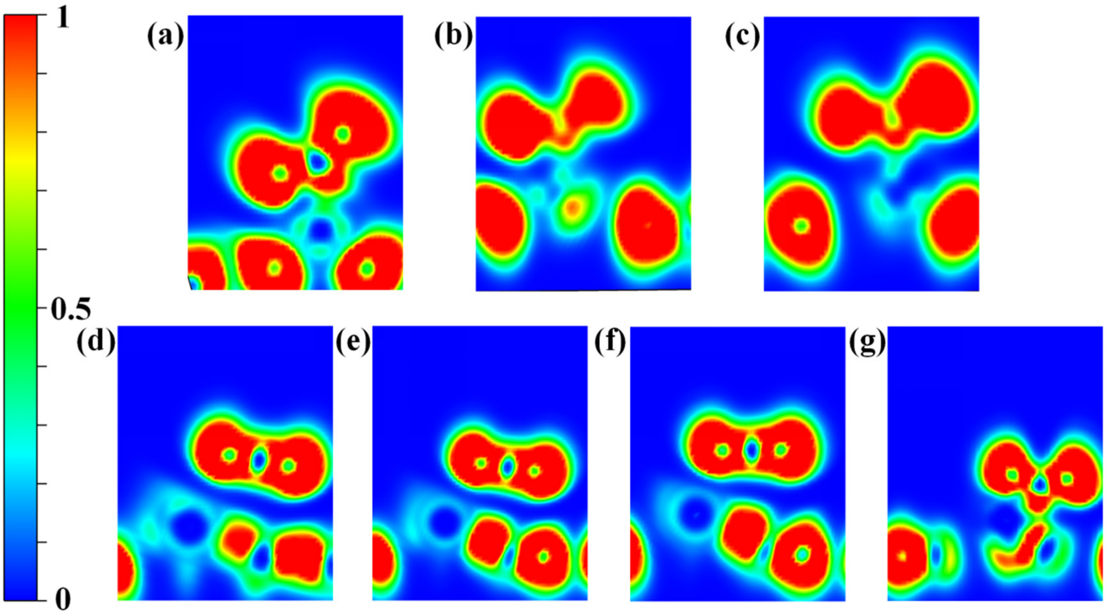

Figure S2 ELF values on the (–1, 1, –1) isosurface of the SCAs: (a) Mn@$B_{-1}N$; (b) Fe@$B_{-1}N$; (c) Co@$B_{-1}N$; (d) Ni@$B_{-1}N$; (e) Cu@$B_{-1}N$; (f) Zn@$B_{-1}N$; (g) Co@BN_{-1}.

ELF values in the range of 0.8 – 1.0 correspond to strongly localized electron regions, 0.6 – 0.8 indicate moderately localized regions, 0.3 – 0.5 represent weakly localized or partially

delocalized regions, and values below 0.3 correspond to highly delocalized electron domains.

Table S3 The number of charges transferred by a monatomic catalyst to CO_2.

	Bader (e^-)
Mn@B_{-1}N	0.536
Fe@B_{-1}N	0.392
Co@B_{-1}N	0.308
Ni@B_{-1}N	0.02
Cu@B_{-1}N	0.014
Zn@B_{-1}N	0.002
Co@BN_{-1}	0.257

Table S4 The number of charges transferred by SACs to H

	Bader (e^-)
Mn@B_{-1}N	0.21
Fe@B_{-1}N	0.12
Co@B_{-1}N	0.33
Ni@B_{-1}N	0.25
Cu@B_{-1}N	-0.36
Zn@B_{-1}N	0.13
Co@BN_{-1}	0.37

Possible path in the CO_2RR process

Path 1:

$$* + CO_2 \rightarrow * CO_2$$
$$* CO_2 + H^+ + e^- \rightarrow * COOH$$
$$* COOH + H^+ + e^- \rightarrow * CO + H_2O$$
$$* CO + H^+ + e^- \rightarrow * COH$$
$$* COH + H^+ + e^- \rightarrow * C + H_2O$$
$$* C + H^+ + e^- \rightarrow * CH$$
$$* CH + H^+ + e^- \rightarrow * CH_2$$
$$* CH_2 + H^+ + e^- \rightarrow * CH_3 + H_2O$$
$$* CH_3 + H^+ + e^- \rightarrow * CH_4$$

Path 2:

$$* + CO_2 \rightarrow * CO_2$$
$$* CO_2 + H^+ + e^- \rightarrow * COOH$$
$$* COOH + H^+ + e^- \rightarrow * CO + H_2O$$
$$* CO + H^+ + e^- \rightarrow * COH$$
$$* COH + H^+ + e^- \rightarrow * CHOH$$
$$* CHOH + H^+ + e^- \rightarrow * CH + H_2O$$
$$* CH + H^+ + e^- \rightarrow * CH_2$$
$$* CH_2 + H^+ + e^- \rightarrow * CH_3$$
$$* CH_3 + H^+ + e^- \rightarrow * CH_4$$

Path 3:

$$* + CO_2 \rightarrow * CO_2$$

$$* CO_2 + H^+ + e^- \rightarrow * COOH$$
$$* COOH + H^+ + e^- \rightarrow * CO + H_2O$$
$$* CO + H^+ + e^- \rightarrow * COH$$
$$* COH + H^+ + e^- \rightarrow * CHOH$$
$$* CHOH + H^+ + e^- \rightarrow * CHHOH$$
$$* CHHOH + H^+ + e^- \rightarrow * CH_2 + H_2O$$
$$* CH_2 + H^+ + e^- \rightarrow * CH_3$$
$$* CH_3 + H^+ + e^- \rightarrow * CH_4$$

Path 4:

$$* + CO_2 \rightarrow * CO_2$$
$$* CO_2 + H^+ + e^- \rightarrow * COOH$$
$$* COOH + H^+ + e^- \rightarrow * CO + H_2O$$
$$* CO + H^+ + e^- \rightarrow * CHO$$
$$* CHO + H^+ + e^- \rightarrow * CHHO$$
$$* CHHO + + H^+ + e^- \rightarrow * CHHOH$$
$$* CHHOH + H^+ + e^- \rightarrow * CH_2 + H_2O$$
$$* CH2 + H^+ + e^- \rightarrow * CH_3$$
$$* CH3 + H^+ + e^- \rightarrow * CH_4$$

Path 5:

$$* + CO_2 \rightarrow * CO_2$$
$$* CO_2 + H^+ + e^- \rightarrow * COOH$$
$$* COOH + H^+ + e^- \rightarrow * CO + H_2O$$
$$* CO + H^+ + e^- \rightarrow * CHO$$
$$* CHO + H^+ + e^- \rightarrow * CHOH$$
$$* CHOH + H^+ + e^- \rightarrow * CH + H_2O$$
$$* CH + H^+ + e^- \rightarrow * CH_2$$
$$* CH_2 + H^+ + e^- \rightarrow * CH_3$$
$$* CH_3 + H^+ + e^- \rightarrow * CH_4$$

Path 6:

$$* + CO_2 \rightarrow * CO_2$$
$$* CO_2 + H^+ + e^- \rightarrow * COOH$$
$$* COOH + H^+ + e^- \rightarrow * CO + H_2O$$
$$* CO + H^+ + e^- \rightarrow * CHO$$
$$* CHO + H^+ + e^- \rightarrow * CHOH$$
$$* CHHO + + H^+ + e^- \rightarrow * CHHOH$$
$$* CHHOH + H^+ + e^- \rightarrow * CH_2 + H_2O$$
$$* CH_2 + H^+ + e^- \rightarrow * CH_3$$
$$* CH_3 + H^+ + e^- \rightarrow * CH_4$$

Path 7:

$$* + CO_2 \rightarrow * CO_2$$
$$* CO_2 + H^+ + e^- \rightarrow * COOH$$
$$* COOH + H^+ + e^- \rightarrow * CO + H_2O$$
$$* CO + H^+ + e^- \rightarrow * CHO$$

$$* CHO + H^+ + e^- \rightarrow * OCHH$$
$$* OCHH + + H^+ + e^- \rightarrow * OCHHH$$
$$* OCHHH + H^+ + e^- \rightarrow * O + CH_4$$
$$* O + + H^+ + e^- \rightarrow * OH$$
$$* OH + + H^+ + e^- \rightarrow * H_2O$$

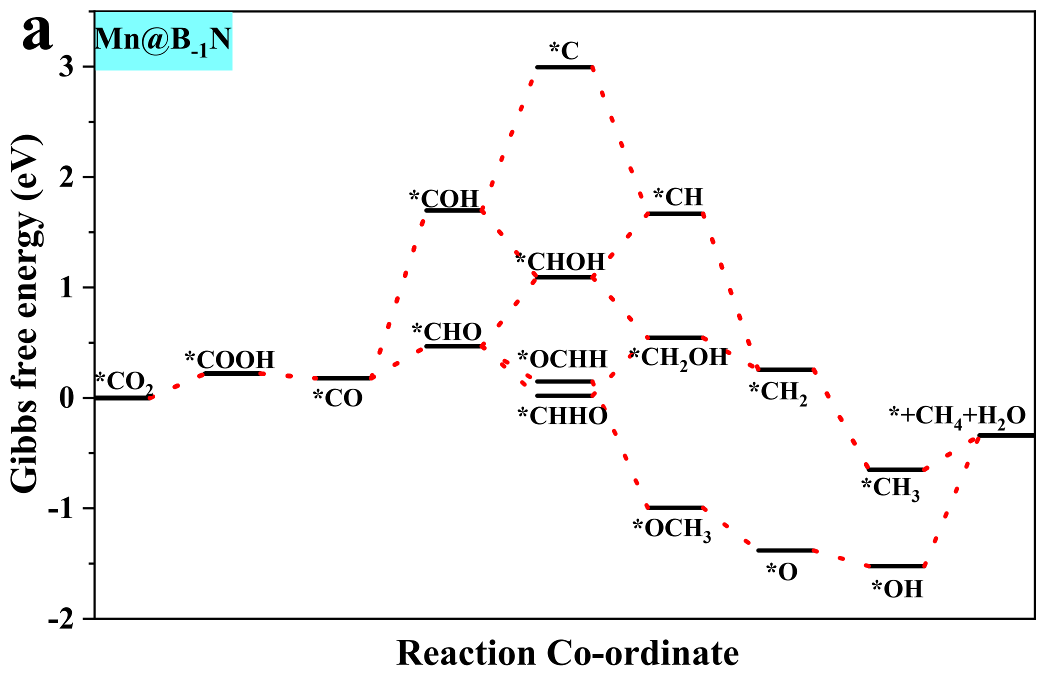

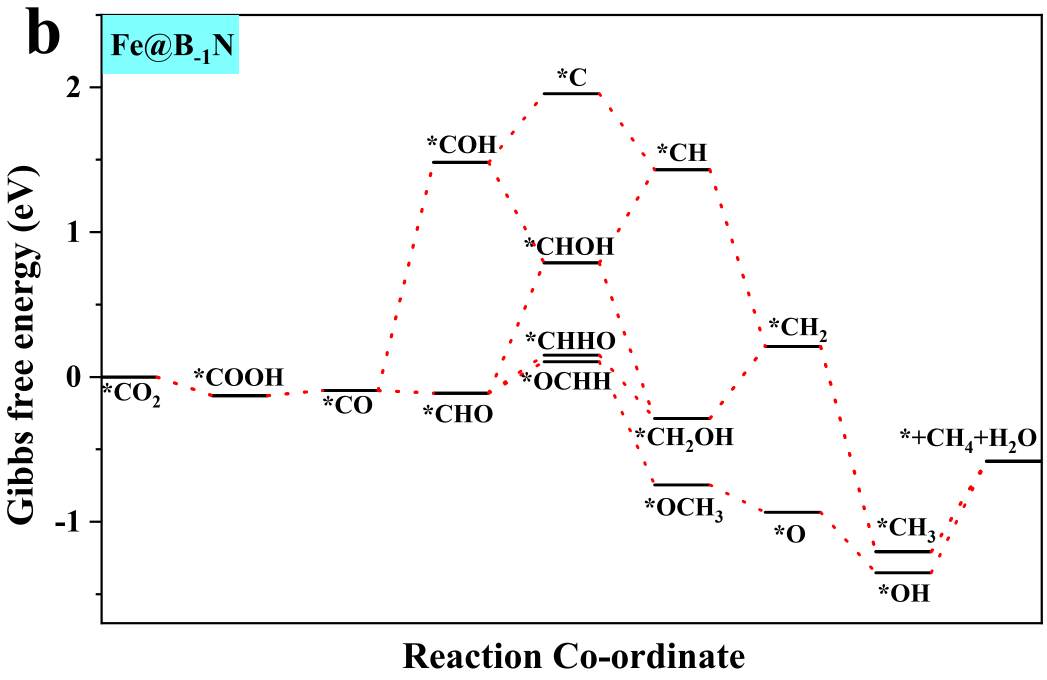

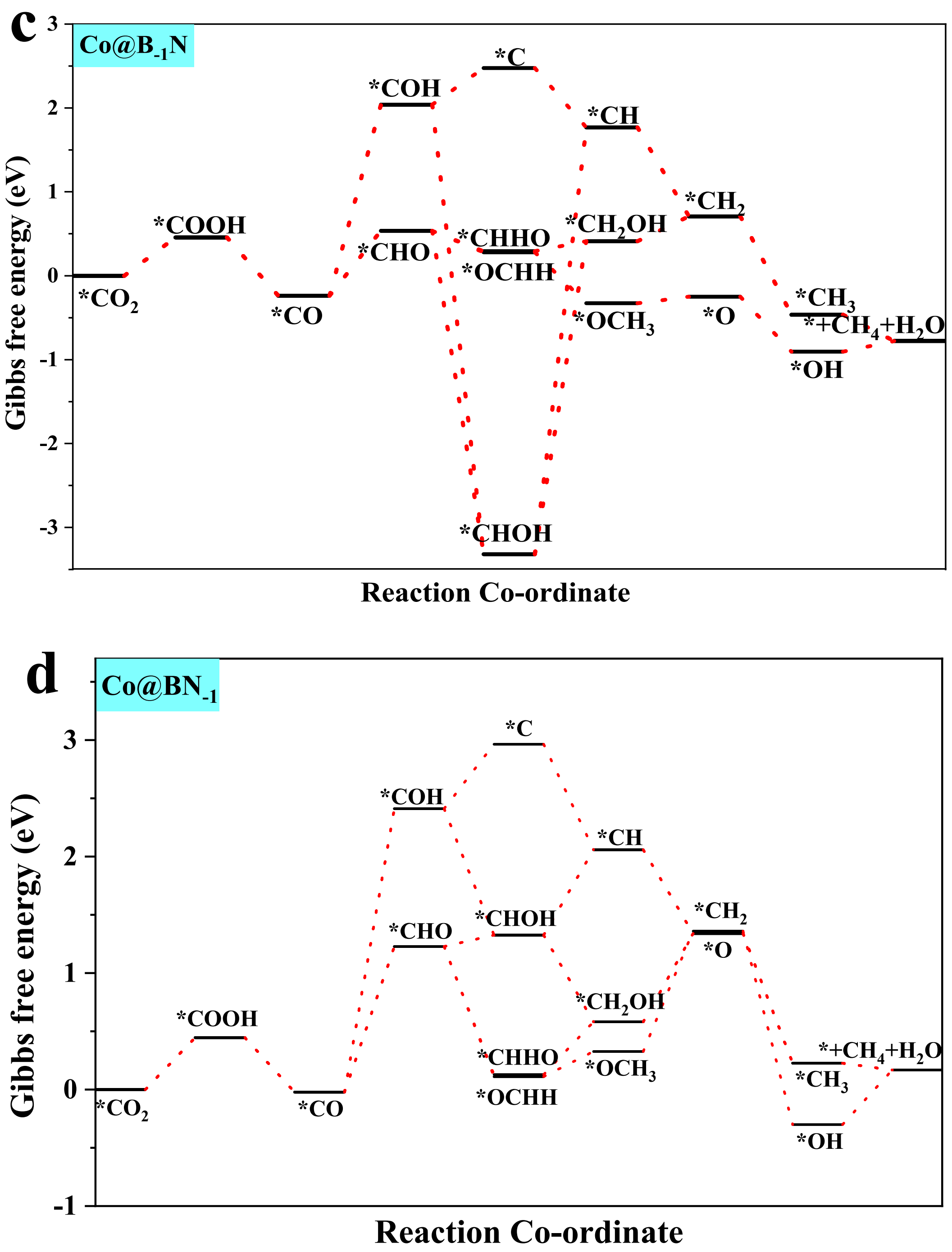

Figure S3 Seven specific reaction pathways for the production of CH_4 through the CO_2RR (Carbon Dioxide Reduction Reaction), Gibbs free energy step diagram of single-atom catalyst CO_2RR: (a) Mn@B_{-1}N; (b) Fe@B_{-1}N; (c) Co@B_{-1}N; (d) Co@BN_{-1}.
